# Control of Crystallization of PBT-PC Blends by Anisotropic SiO_2_ and GeO_2_ Glass Flakes

**DOI:** 10.3390/polym14214555

**Published:** 2022-10-27

**Authors:** Björn Düsenberg, Julian D. Esper, Felix Maußner, Magdalena Mayerhofer, Jochen Schmidt, Wolfgang Peukert, Andreas Bück

**Affiliations:** 1 Institute of Particle Technology, Friedrich-Alexander-Universität Erlangen-Nürnberg, Cauerstraße 4, D-91058 Erlangen, Germany; 2 Collaborative Research Center 814—Additive Manufacturing (DFG, German Research Foundation), Am Weichselgarten 9, D-91058 Erlangen, Germany

**Keywords:** polymer-blends, crystallization modifiers, co-comminution, functional filler, glass flakes, anisotropic particles

## Abstract

Polymer composites and blend systems are of increasing importance, due to the combination of unique and different material properties. Blending polybutylene terephthalate (PBT) with polycarbonate (PC) has been the focus of attention for some time in order to combine thermo-chemical with mechanical resistance. The right compounding of the two polymers is a particular challenge, since phase boundaries between PBT and PC lead to coalescence during melting, and thus to unwanted segregation within the composite material. Amorphization of the semi-crystalline PBT would significantly improve the blending of the two polymers, which is why specific miscibility aids are needed for this purpose. Recent research has focused on the functionalization of polymers with shape-anisotropic glass particles. The advantage of those results from their two-dimensional shape, which not only improves the mechanical properties but are also suspected to act as miscibility aids, as they could catalyze transesterification or act as crystallization modifier. This work presents a process route for the production of PBT-PC blends via co-comminution and an in-situ additivation of the polymer blend particles with anisotropic glass flakes to adjust the crystallinity and therefore enhance the miscibility of the polymers.

## 1. Introduction

Polymer blends are multi-materials consisting of a predominantly present polymer, which is often used as a matrix, and one or more materials, which are continuously or discontinuously dispersed within [1]. The properties of the blend system depends on several parameters, e.g., the matrix polymer, the dispersed polymer, the dispersion inside the matrix polymer, the effects at the polymer and the spatial arrangement of the materials with respect to each other [2,3] as well as on the properties of further filler materials within the blend.

The possibilities for customized blends are manifold and range from the modification of mechanical properties to the adjustment of electrical and thermal conductivity, which makes composite development and production an interesting area for both science and industry [4,5,6,7]. Out of this interest a variety of several new and potential blends for industrial applications has been developed in recent years. This is due to the wide margin of superior properties compared to conventional materials [8,9,10,11]. These include in particular polymer combinations, which often provide a higher strength-to-weight ratio, simple product property customization, flexible manufacturing processes and lower cost. 

Filled polymer systems exhibit a different process behavior compared to their neat polymer counterparts [12,13]. Additives are often used in polymer formulations, to improve specific properties such as the powder flowability [11,14] or to adjust the layer hardness of the component after a powder coating process [15,16]. However, a rather negative property of most fillers is that they influence the crystallization behaviour of the polymers. This results from additives (oftentimes nano particles) that serve as crystallization nuclei at which crystal growth can take place at an accelerated rate [17,18,19,20,21].

Polycarbonate (PC) and polybutylene terephthalate (PBT) blends [22,23] have been previously investigated, due to their unique properties. PBT, has a high thermal and mechanical stability, as well as a low absorbtion of water [24,25] whereas PC has a significant hardness and dimensional stability [26,27,28]. It was shown by Pompe et. al. [22] that the miscibility of PBT-PC and thus the mixture of properties depends on the amorphous portion of the PBT. The effect of high miscibility of amorphous components is well known in pharmaceutical formulations, [29,30] where amorphous solid dispersions are created as drug delivery systems but rarely used with technical polymers as their properties often prevent miscibility on a molecular level. To make use of a higher miscibility and therefore enhance polymer blend systems, it is important to find advanced functional additives that favour the effect of amorphization. 

PBT-PC blends are usually reinforced with further materials like talc [31,32] or glass fibers/spheres [33,34,35] which are enhancing the mechanical properties but are not suitable as miscibility aid. Lately, (glass) flakes [8,36,37] have been utilized as a filling material to selectively enhance desired properties in polymers or coatings. Flake-like glass particles are characterized as lamella structures, unlike e.g., glass fibers and exhibit high aspect ratios with lateral dimensions well in the micron range while being just several hundred nanometers thick [37,38,39]. This unique shape can increase fire resistance or enhance the mechanical properties due to improved structural stability, which is why flake like structures are useful as additive in the first place. As glasses are observed to enhance the amorphous partition in polymer blends, [40,41,42] the next step is to combine the flake like structures with the miscibility enhancing properties. The use of glass flakes for control of the crystallinity has not been addressed, yet.

In this study, flakes of three different glasses, namely vitreous SiO_2_, SiO_2_-GeO_2_ and, for the first time, vitreous GeO_2_ are evaluated as crystallization modifier. SiO_2_ is an often used material to functionalize polymers, especially as nano particulate filler or to enhance the flowability [9,14]. Therefore the functionalization with this material as anisotropic flake is ideal to compare the influence of the form on thermal properties. The usually mechanically more fatigue resistant [43] GeO_2_ was chosen to investigate the influence of the material type because, like SiO_2_, it has a tetragonally arranged structure. GeO_2_ glasses are often used in special applications such as fibre optics, as a catalyst, and e.g., infrared optical systems [44] or in newer work in battery anodes [39,45]. All types of glass flakes are produced by wet-comminution and combined with co-comminuted PBT-PC, in a stirred media mill. It is investigated how the glass type and filler concentration acts as crystallization adjustor for the practically important PBT-PC blend, and therefore improve material and process understanding.

## 2. Materials and Methods

### 2.1. Wet Comminution

Wet (co-)comminution in stirred media mills (SMM) is used to produce the PBT-PC blends by intermixing them into each other as well as producing the anisotropic flakes. It is called co-comminution, when two or more materials, instead of one material, are comminuted and simultaneously intermixed (blended) in one process step. The advantage is that the particles can not only be comminuted and thus brought to target size, but at the same time the materials are kneaded into each other [8,46,47]. Co-comminution is possible via dry milling techniques [8], dry and cryogenic [48] and with wet using SMM. The process challenge is that PBT and PC are comminuted at different rates. Comminution in SMMs was described by Kwade [49], where the main quantities to assess the process function are stress energy *SE_max_* and stress number *SN*. *SE_spec_* is defined as the mass specific energy as shown in Equation (1).

(1) $$SE_{spec}\propto \;SE_{max}\times SN$$

The maximum stress energy *SE_max_*, which can be transferred to the material during a collision of the grinding beads is defined as the maximum kinetic energy of the grinding beads [50,51]:

(2) $$SE_{max}=d_{GM}^{3}\times v_{tip}^{2}\times \rho_{GM}\times \phi =d_{GM}^{3}\times v_{tip}^{2}\times \rho_{GM}\frac{E_{GM}}{E_{mat}+E_{GM}}$$

with *d_GM_* the grinding bead diameter, *v_tip_*: the stirrer speed at the tip, *ρ_GM_* the density of the grinding beads, *E_GM_* the Young’s modulus of the grinding media and *E_mat_* Young’s modulus of the feed material. This model takes the Young’s modulus of the stressed material and the grinding media into account, whereby when grinding polymers the factor $\frac{E_{GM}}{E_{mat}+E_{GM}}$ is close to unity [52]. For co-comminution processes, there is currently no equation to calculate *SE_max_*. Therefore, *SE_max_* for single- materials, as given in Equation (2), is currently the best way to approximate the energy input into the material during stressing in a SMM. 

### 2.2. Polymers

PBT and PC (both Resinex GmbH, Zwingenberg, Germany) were used. The powder was presieved to eliminate the particle sizes >500 µm and therefore unify the PSD of the educts. The initial properties of PBT and PC are shown in Table 1.

### 2.3. Glasses

Three different glass compositions were used for the presented experiments, which were produced from SiO_2_ (Heraeus Suprasil^®^, Hanau, Germany) and crystalline GeO_2_ (>99.99%; Merck, Darmstadt, Germany). The SiO_2_-GeO_2_ and GeO_2_ glasses were prepared by mixing the appropriate amount of feed materials and mixing it in a tumbling mixer (T2F, Willy A. Bachofen AG, Muttenz, Switzerland) for 30 min to ensure a homogeneous powder mixture. Melting was performed in a muffle oven at 1500 °C for 3 h. Heating ramp was set to 100 °K min^−1^. The glasses were quenched between 2 brass plates, crushed to a fine powder with pestle and mortar and remelted to ensure a homogeneous distribution of SiO_2_ and GeO_2_ throughout the glass sample. In this work SiO_2_ glass (100% purity), GeO_2_ glass (100% purity) and a 50:50 mixture of both will be used.

**Figure 1:**
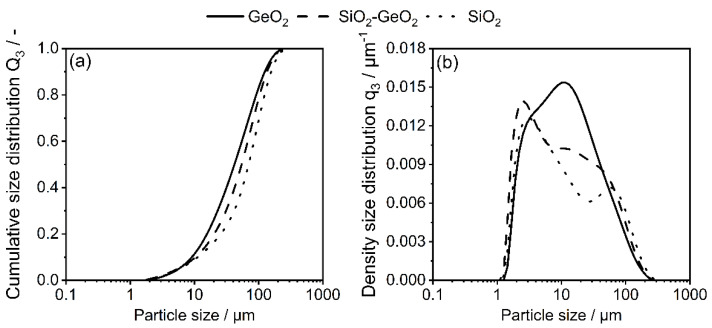
(**a**) Cumulative particle size distribution and (**b**) density particle size distribution of glass.

All bulk glass materials were crushed with pestle and mortar and sieved to a size below 125 µm prior to the comminution experiments inside SMM. Cumulative particle size distributions Q_3_ of the glass feeds where measured via laser diffraction and are showcased in Figure 1. 

All pre-crushed glasses exhibited a similar particle size distributions and particulate characteristrics of the glasses, such as x_10,3_, x_50,3_, x_90,3_, mass-specific surface area, Sauter diameter and powder density, are given in Table 2.

The used grinding solvent for the shape formation in a stirred-media-mill (PE 075, Netzsch-Feinmahltechnik GmbH, Selb, Germany) was 1-pentanol (analytical grade, Merck, Darmstadt, Germany) as reported in our earlier work [38,39,53,54]. The choice of solvent is a key process parameter to yield flake like particles rather than irregularly shaped comminution products. After grinding and stressing in 1-pentanol, all glass powder samples were washed in ethanol and subsequently dried at 80 °C. Any residual surface organics were removed for at 300 °C for 2 h. Table 3 shows the experimental conditions of the glass comminution process in a SMM.

### 2.4. Composite Formation by Co-Comminution in a Stirred Media Mill

Co-grinding of the polymers is carried out by a stirred-media-mill PE 075 (Netzsch-Feinmahltechnik GmbH, Selb, Germany), equipped with a ceramic grinding chamber and an eccentric 3-disk ceramic agitator with a frequency of 2000 min^−1^ (stirrer speed = 6.5 m s^−1^). The grinding chamber is tempered to 15 °C by the unistat 905 cryostat (Huber Kältemaschinenbau AG, Offenburg, Germany). Spherical, Yt-stabilized ZrO_2_ grinding media (YTZ, Tosoh Inc., Tokyo, Japan) with a diameter of 2 mm are used. Ethanol 96% (VWR Chemicals, Radnor, PA, USA) serves as the suspension liquid. Co-grinding takes a total of 7 h, with the glass flakes being added after 6 h to be incorporated into the PBT-PC blend. The powder is separated from the liquid by vaccum flitration and a Büchner funnel (Grade 1 filter, Whatman, Maidstone, UK). The paste is dried at 110 °C in an oven (Heraues instruments, Hanau, Germany) afterwards for 12 h. All parameters are listed in Table 4. 

The produced formulations are listed in Table 5. Each formulation is produced three times to ensure reproducibility.

### 2.5. Characterization Methods

#### 2.5.1. Scanning Electron Microscopy

The polymer particles have been characterized by scanning electron microscopy (SEM) using a Gemini Ultra 55 (Zeiss, Jena, Germany) device equipped with a Everhart-Thornley (SE2) (Zeiss, Jena, Germany) detector. An acceleration voltage of 1 kV has been applied to the particles. Morphological characterization of the glass flakes was done at an accelerating voltage of 1.8 kV. Glass samples were prepared for characterization by dispersing particulates in ethanol and suspending single droplets on a Si wafer. The polymer blends and the polymer-glass blends are prepared by deposition on a carbon sticky pad, which is placed on the sample holder.

#### 2.5.2. Thermal Analysis

The thermal behaviour of the powders was measured by differential scanning calorimetry (DSC) to analyze the influence of the glass flakes on the crystallization and crystallinity. For this purpose a Polyma 214 (Netzsch, Selb, Germany) was used. The samples (weighing 10 mg ± 0.1 mg) were measured in covered aluminum pans (Concavus Lids (Al), NGB817526, Netzsch, Selb, Germany) and measured with dry nitrogen gas, purging at 40 mL min^−1^. The measurement program consists of the following steps: (1) heating from 20 °C to 300 °C at 10 K min^−1^, (2) isothermal hold time of 1 min, (3) cooling from 300 °C to 20 °C at 10 K min^−1^, (4) isothermal hold time of 1 min. The measuring program is executed twice. For the analysis of the DSC thermgramms, the software “Proteus Analysis” (Netzsch, Selb, Germany) was used. The evaluated peak width is defined as the distance between the onset and the offset of a peak at the hight of the baseline. The relative crystallinity is evaluated by use of the melting enthalpy of the formulations. For this, the crystallinity of PBT-PC is used as the 100% standard (examples of the measured DSC curves can be found in the Supplementary Materials Figures S4–S6). 

#### 2.5.3. Helium Pycnometry

True glass flake powder density was determined via a helium pycnometer (AccuPyc 1330, Micromeritics, Norcross, GA, USA). All washed and dried powder samples were kept at 60 °C prior to this analysis. The reported values are means of three separate and independent measurements.

#### 2.5.4. Volumetric Nitrogen Sorption Analyzer

Nitrogen sorption measurements at liquid nitrogen temperature were performed using a volumetric gas sorption analyzer (Nova 4200e, Quantachrome, Boynton Beach, FL, USA). Mass-specific surface areas were determined from dried and degassed samples (2 h at 200 °C under vacuum) according to the BET (Brunauer-Emmett-Teller) method (cf. Table 2) [55]. The pressure range (0.02–0.35) was divided into 8 data points (BET data is available in the Supplementary Materials Figure S3).

#### 2.5.5. Fourier Transform Infrared Spectroscopy

Fourier transform infrared spectroscopy (FTIR) spectra were recorded using a FTS 311 spectrometer (Varian, Palo Alto, CA, USA) in the range of 1600–400 cm^−1^ with a spectral resolution of 4 cm^−1^. Suitable powder samples for the measurement in transmission geometry were prepared by mixing and tableting approximately 300 mg dry KBr (UVASol, Merck, Darmstadt, Germany) with 1 mg of the sample. The recorded spectra data was subjected to mathematical smoothing (7–Point–Savitzky–Golay) and normalization on the maximum value. Occuring vibrational modes were matched with modes from available published works [56,57,58,59,60,61,62,63,64].

#### 2.5.6. Particle Size Analysis

Particle size analysis is performed via laser diffraction using Mastersizer 2000 equipped with a Hydro 2000 wet dispersing unit (both Malvern Panalytical, Malvern, UK). To enhance the wettability and therefore enhance the measurement the powders were dispersed in ethanol (96%, denatured). Furthermore, sodium dodecyl sulfate (SDS, Merck, KGaA, Darmstadt, Germany) was added to the reservoir of the Mastersizer. A stirring rate of 3500 rpm and ultrasonication at 50% power were set at the measurement device. 

## 3. Results and Discussion

### 3.1. Shape Formation of Glasses Inside a Stirred Media Mill

**Figure 2:**
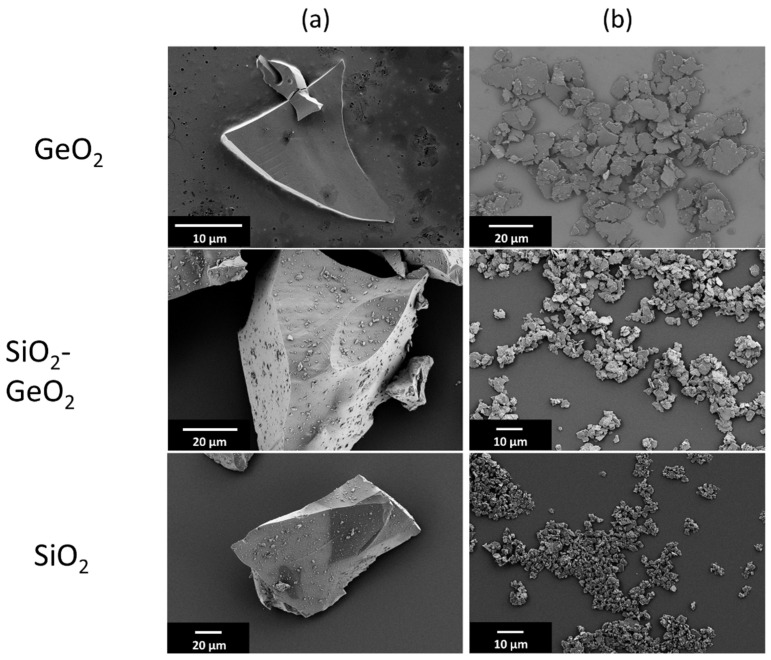
Glass after pre-crushing (**a**) before the comminution step and comminuted glass flakes (**b**).

Previous works have reported on the brittle-to-ductile transition of vitreous glass particles below a certain particle size. Figure 2 showcases SEM micrographs of the different pre-crushed glasses after melt-quenching (left column) and their corresponding particles after stressing in a stirred media mill for 5 h at a stirrer speed of 6.5 m s^−1^ using 1.0 mm grinding beads (right column). The glasses show irregular particles with sharp edges due to the brittle breakage during the pre-crushing with pestle and mortar. All stressed glasses exhibit a flake-like particle shape with smooth surfaces, high aspect ratios and lateral dimensions well in the micron range. From SEM image analysis, it is clear, that the shape formation and therefore ductile behavior of the glasses also holds true for SiO_2_-GeO_2_ and GeO_2_ glasses.

The change in density (Supplementary Materials Figure S1a) and surface area increase (Supplementary Materials Figure S1b) are increasing with processing time. Due to the shape anisotropic nature of the resulting particles, size comparison is quite difficult. Therefore, the surface-equivalent spherical diameter, Sauter diameter *d*_3,2_, was determined via Equation (3):

(3) $$d_{3,2}=\frac{6000}{S_{m}\times \rho }$$

**Figure 3:**
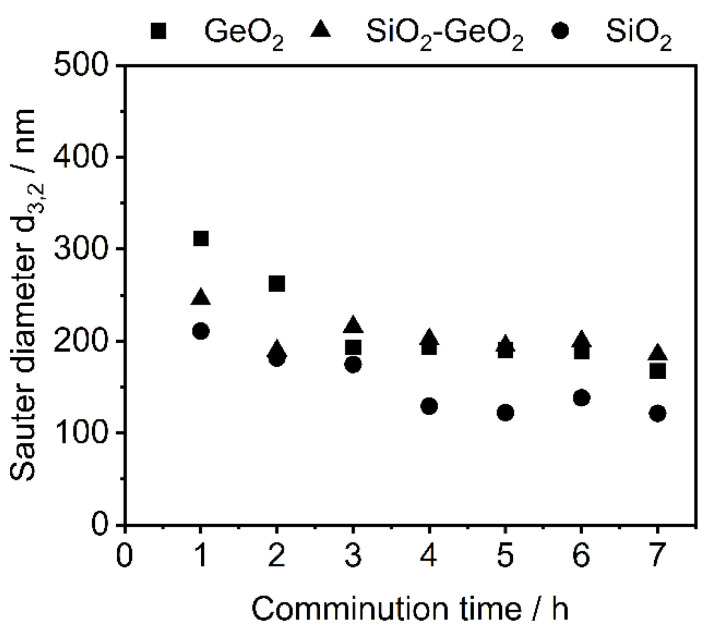
Evolution of the surface-equivalent spherical particle diameter *d*_3,2_ (Sauter diameter) with increasing comminution time.

The Sauter mean diameter *d*_3,2_ is defined as the diameter of a spherical particle that exhibits the sample volume/surface area ratio as the measured shape-anisotropic particle and is commonly used to describe and compare non-spherical particle systems. With $S_{m}$ as the mass specific surface area obtained by the BET measurement. In the presented Sauter diameter data, a strong size reduction can be observed during first 3 h for all glasses, which slows down after that. No substantial change in terms of the Sauter diameter is observed after 5 h of stressing (Figure 3).

**Figure 4:**
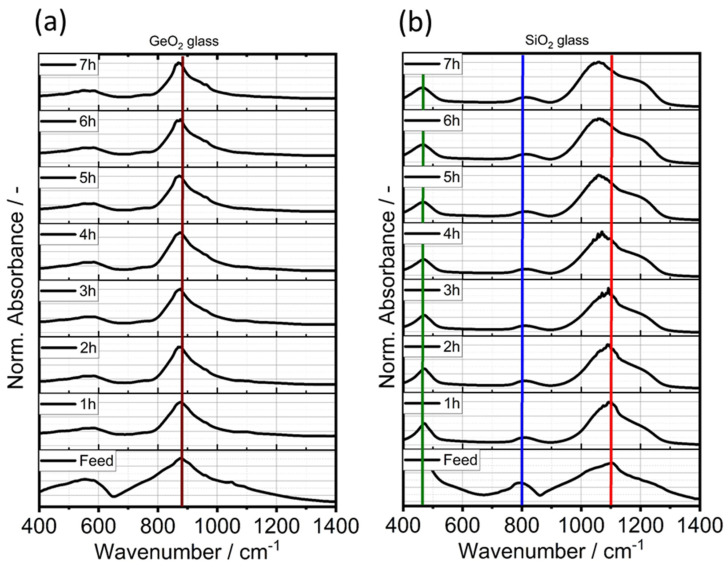
IR spectra evolution of GeO_2_ glass (**a**) and SiO_2_ glass (**b**) with increasing comminution time. The colored lines correspond to the absorption bands displayed in Table 6.

**Figure 5:**
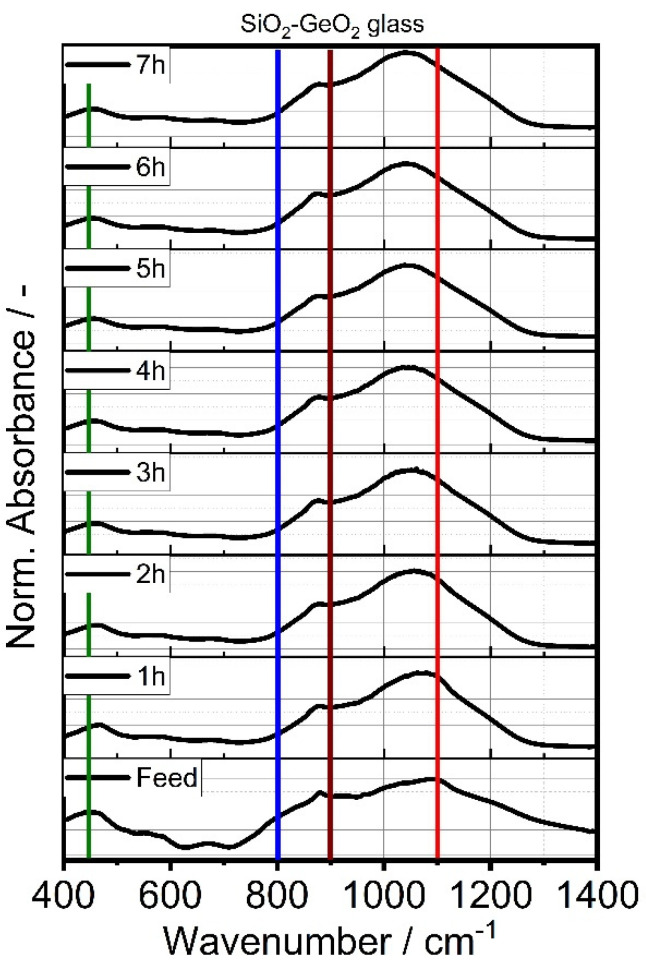
IR spectra evolution of the mixed SiO_2_-GeO_2_ glass with increasing comminution time. The colored lines correspond to the absorption bands displayed in Table 6.

From Sauter diameter *d*_3,2_ analysis, a particle size reduction was observed for the first 4 h of comminution. Further stressing did not lead to any significant size reduction of the glasses and suggests plastic deformation due to mechanical energy input, rather than brittle breakage. Possible structural changes of the stressed glasses were investigated in terms of Fourier transform IR spectroscopy. A similar result can be observed from the derived IR spectroscopy data. Figure 4 and Figure 5 showcases the IR spectra of GeO_2_ glass (a) and SiO_2_ glass in the wavenumber range of 400–1400 cm^−1^ with their corresponding spectra with increasing comminution time. The spectra were normalized using the maximum absorption value of the respective glass system. Table 6 showcases the IR-bands evaluated in this study.

GeO_2_ glass shows one significant peak absorption band peak at ~890 cm^−1^ which can be assigned to asymmetric vibration of the Ge-O-Ge bond [59] and a smaller, less pronounced peak at 450 cm^−1^ that is characteristic for the Ge-O-Ge bending vibration. The observed spectrum of the GeO_2_ feed glass is in good agreement with previously reported GeO_2_ glass spectra [58,59,66]. The absorption band at ~580 cm^−1^ decreased with increasing comminution time. For SiO_2_, the strong 1100 cm^−1^ absorption band exhibits small additional shoulders at 1050 cm^−1^ and 1200 cm^−1^ that can all be assigned to the Si-O-Si stretching vibration. The observed shoulder is visible in all samples, but increases with production time in the SMM, therefore another reason can be an increase in Si-OH groups, resulting from the production in 1-pentanol. This effect is the opposite of the observed decreasing shoulder when calcinating silica glass [68,69]. Furthermore, pronounced absoprtion bands can be observed at 800 cm^−1^ and 460 cm^−1^. Bell and Hibbins Buttler were the first to assign these smaller absorption bands to the symmetric and asymmetric vibrational bands of Si-O-Si, respectively [70]. Table 6 lists the main vibrational absorption bands for SiO_2_- and GeO_2_-based glasses and their corresponding wavenumbers.

**Figure 6:**
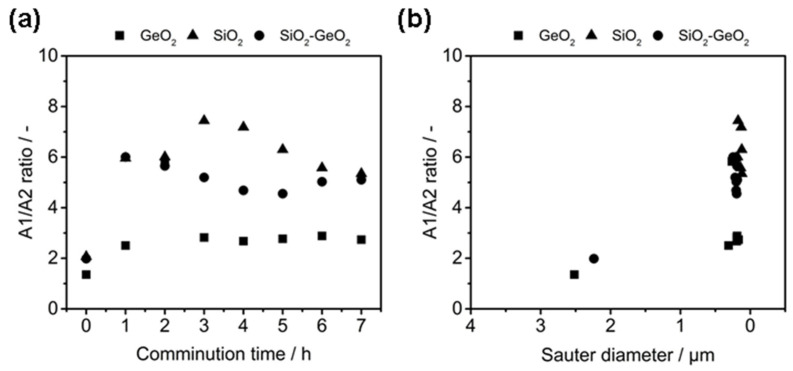
A1/A2 ratio as a function of comminution time (**a**) and Sauter diameter (**b**).

Figure 6 showcases the A1/A2 ratio against the Sauter diameter and comminution time, where A1 and A2 correspond to the absorption of the 1100 cm^−1^ and 800 cm^−1^ bands of the SiO_2_ glass, respectively according to the method first published by Iwao [66]. The A1/A2 ratio represents the proportion of asymmetric stretch vibration to symmetric stretch vibration of Si-O-Si, which can be considered as a measure of structural disorder. In case of the pure GeO_2_ glass, the peaks A1 and A2 refer to the absorption bands observed at 550 cm^−1^ and 870 cm^−1^, which can be assigned to the asymmetric stretch vibration and sym-metric stretch vibration of the Ge-O-Ge bond, respectively. All glasses show, that the A1/A2 ratio is significantly increased from a value of ~2 of the feed glasses to more >6 for the SiO_2_ glass flakes and to ~3 for GeO_2_ glass flakes, which represents a significant struc-tural deformation of the homogeneous glasses. The mixed SiO_2_-GeO_2_ glass displays a mixed behavior of both glasses, which can be seen as a good indication, that both feeding materials formed a homogeneous glass mixture during melting and resulting in a glass with mixed properties of both glass forming constituents.

From the given results, stressing of the glasses was stopped after 5 h and the products were used for all further experiments.

### 3.2. Co-Comminution and Blend Formation

**Figure 7:**
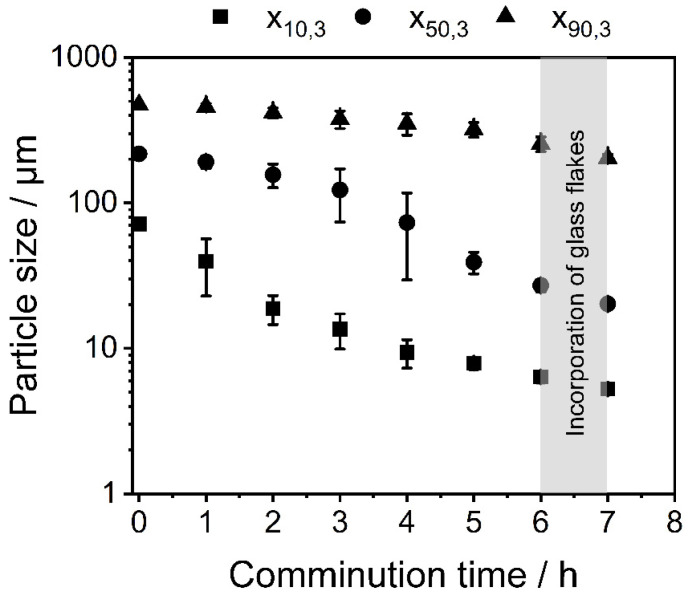
Change in x_10,3_, x_50,3_ and x_90,3_ during co-comminution and formation of the PBT-PC-glass composite.

Co-comminution (blending) of PBT-PC takes place in the first 6 h of the process. It is observed that the particle size after 6 h of co-comminution is x_10_ = 6.3 µm ± 0.6 µm, x_50_ = 27 µm ± 2.5 µm and x_90_ = 254 µm ± 30 µm (Figure 7). Since the purpose was rather to knead the polymers into each other to form a blend system. After 6 h the glass flakes are added to the SMM with the PBT-PC and further size reduction occurs as well as the incorporation of the glass flakes into the polymer matrix as discussed later The final particle size of the PBT-PC glass composite is x_10_ = 5.0 µm ± 0.2 µm, x_50_ = 20 µm ± 1.2 µm and x_90_ = 203 µm ± 18 µm.

**Figure 8:**
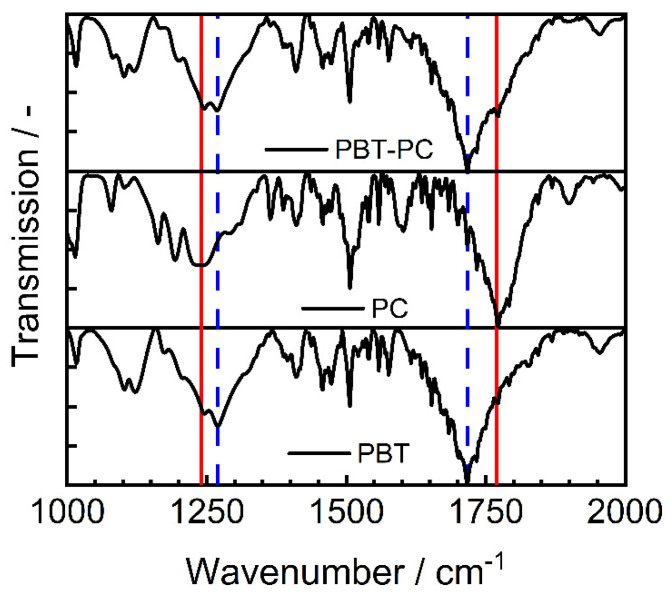
Proof of the blending of PBT and PC during the co-comminution process; PC (red, straight line): O-C-O at 1235 cm^−1^, C=O at 1770 cm^−1^; PBT (blue, dashed line): O-C-O at 1260 cm^−1^, C=O at 1718 cm^−1^ [61,62,63,64].

The ratio of the polymers is confirmed after 6 h by FTIR measurements of the dried powder (see Figure 8). In the composite, both the specific PC bands (red, straight line) at 1235 cm^−1^ (O-C-O bond) and 1770 cm^−1^ (C=O bond) and the specific PBT bands (blue, dashed line) at 1260 cm^−1^ (O-C-O bond) and 1718 cm^−1^ (C=O bond) can be seen [61,62,63,64].

**Figure 9:**
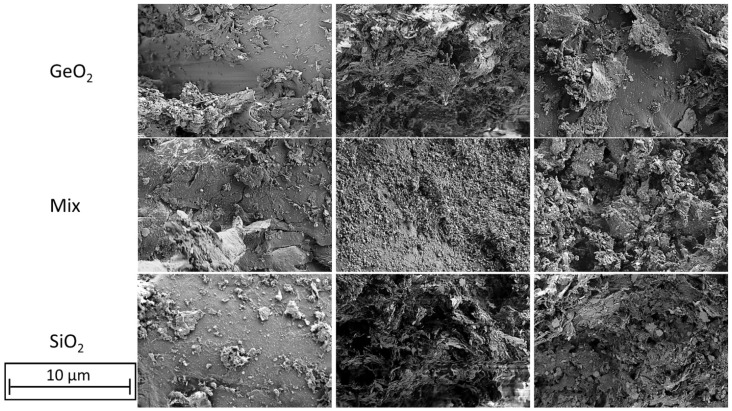
SEM-Images of different formulations. left row 0.1 wt. % glass content; middle row 0.5 wt. % glass content; right row 1.0 wt. % glass content–glass flakes are randomly intermixed with the polymer blends.

The glass flake particles can be found on top of the polymer blend particles and partially embedded into them. This can be seen in Figure 9 at selected filler concentrations of 0.1 wt. %, 0.5 wt. % and 1.0 wt. %. The glass flake spiked blends do not differ from each other on the basis of the glass type.

The possible recycling of co-comminuted polymer blends on this scale is hardly possible at the current state of the art [71]. Especially since the co-comminution is used to make the two polymers inseparable. A possible further use at the end of the life cycle could be re-melting and processing, provided that the resulting crystallinity is sufficient for further use, or using the plastic as a filler.

**Figure 10:**
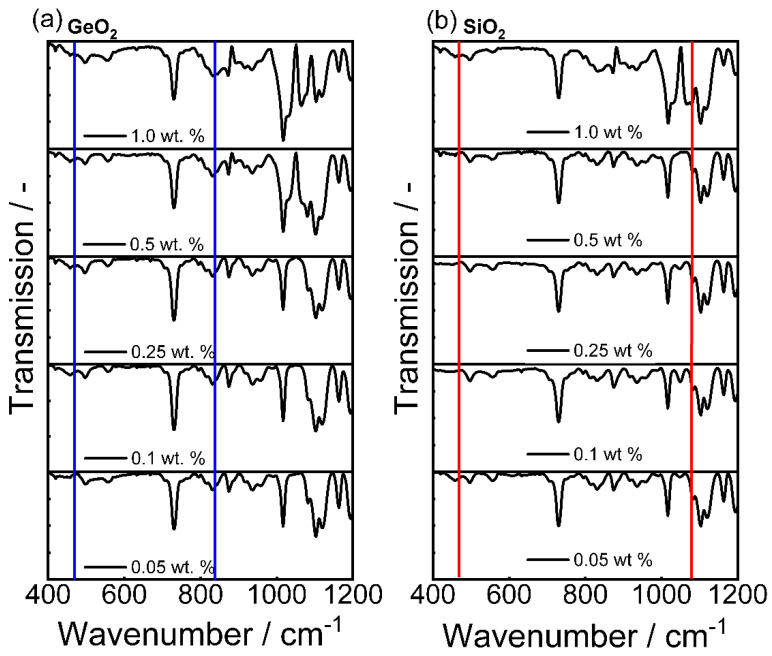
IR spectra of the different PBT-PC-glass blends; (**a**) with pure GeO_2_; (**b**) with pure SiO_2_. Bands are taken from Table 6.

Looking at the normalized IR spectra of the PBT-PC-glass blends (Figure 10), we see both the increase in the GeO_2_ bands (wavenumber wavenumbers; 470 cm^−1^ and 820 cm^−1^, corresponding to Table 6) and the increase in the SiO2 bands (wavenumbers; 450 cm^−1^ and 1070 cm^−1^ corresponding to Table 6). Therefore, the blend formation consisting of PBT-PC and the pure glasses was successful. The measurements additionally confirm the presence of the glasses in all blends. However, due to the small sample quantity, no conclusion can be drawn about the homogeneity of the distribution.

**Figure 11:**
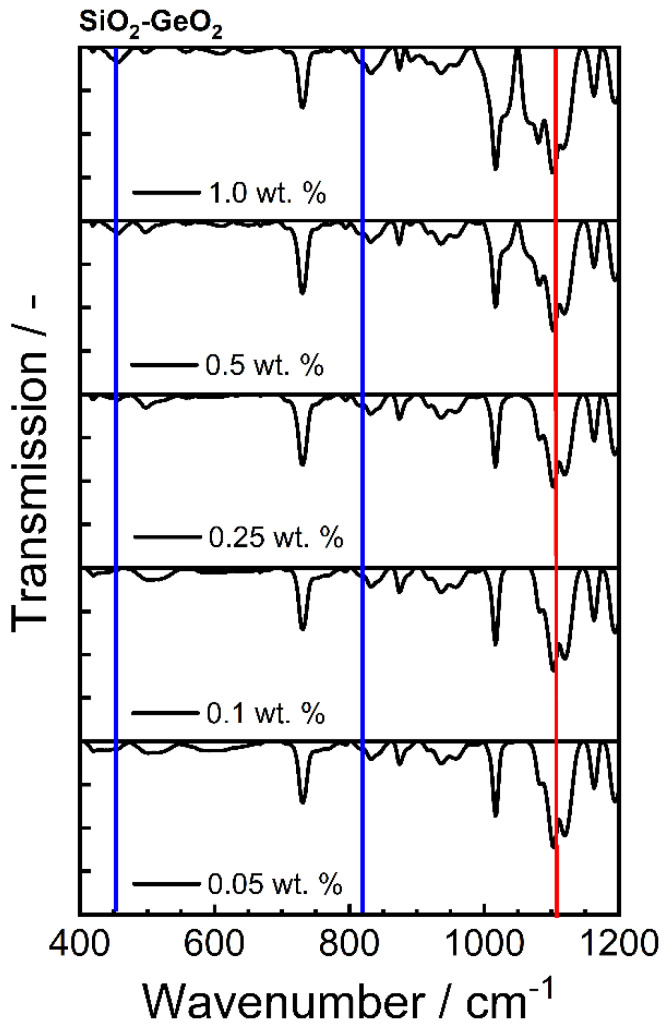
IR spectra of the different PBT-PC-SiO_2_-GeO_2_ blends. Bands are taken from Table 6, blue represents GeO_2_ bands and red represents the SiO_2_ band.

The normalized IR spectra of the PBT-PC-SiO_2_ (Figure 11), we see both the increase in the GeO_2_ (blue; wavenumbers; 470 cm^−1^ and 820 cm^−1^) bands and the increase in the SiO_2_ (red, wavenumber 1070 cm^−1^) bands, which also confirms the blend formation with the glass mixture. 

### 3.3. Influence on the Crystallization and Crystallinity

**Figure 12:**
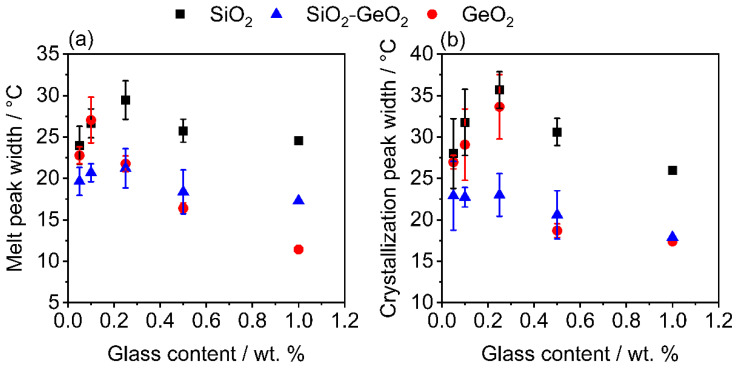
Change in peak width of the different blends; (**a**) melt peak width; (**b**) crystallization peak width.

As two polymers, with different glass transition temperatures are used in this work, the existence of several amorphous phases was evaluated, but different amorphous fractions, as in [72] could not be found in the DSC data (cf. DSC curves in Supplementary Materials S6). After the blend formation, the influence of the glass flakes on the thermal properties of the blends was investigated using DSC. Figure 12a shows the change in melt peak width (MPW) immediately after blending. While SiO_2_ has a relatively constant melt peak width, the MPW changes significantly when the GeO_2_ content is increased. The area of the melting peak describes the enthalpy required to melt the substance, from which the relative crystallinity of the substance (or blend) can be derived. The counterpart to the enthalpy of fusion is the enthalpy of crystallization. If both values are equal, it can be assumed that the crystallinity remains constant. If the enthalpy of crystallization is lower, the crystallinity of the investigated substance decreases—unless post-crystallization occurs—which is why the melting peak is used to determine the crystallinity of the sample. The widths of the peaks indicate whether a substance has inhibitions to melting or crystallization, which can be used to deduce how the heat transport into the product takes place and thus whether melting or crystallization is faster. 

The GeO_2_ reduces the melt peak width by almost 50%. The melting peak width of the SiO_2_-GeO_2_ is located approximately in the middle of the pure substances. 

Figure 12b shows the corresponding crystallization peak widths (CPW). Again, the peak widths are highest for SiO_2_, whereas the CPW of SiO_2_-GeO_2_ and pure GeO_2_ are more similar, especially at additive contents of 0.5 wt. % and higher. As discussed in the presentation of the MPW, a strong variance in the glass content, especially with the very small glass contents, of the DSC samples is to be assumed. 

The results of the decrease in both, melting peak width and crystallization peak width, can be explained by several effects. First reason are the different thermal conductivities of the glasses. It is known [73] that the thermal conductivity of SiO_2_ increases significantly with the addition of GeO_2._ Not only increases GeO_2_ the thermal conductivity, it also has lower specific heat capacity (c_p_) in comparison with SiO_2_ that means, that much less thermal energy is needed to increase the temperature of GeO_2_ and therefore the thermal energy is available to heat/cool down the polymer composite. The specific heat capacity of GeO_2_ (c_p,GeO2_ ~0.5 J g^−1^ K^−1^) [74,75] whereas the specific heat capacity of SiO_2_ (c_p,SiO2_ ~0.72 J g^−1^ K^−1^) [76], which leads to an averaged specific heat capacity of the SiO_2_-GeO_2_ mixture of c_p,SiO2-GeO2_ ~0.6 J g^−1^ K^−1^. These effects in combination are reducing the peak width as is observed in Figure 12. 

The last reason, for the reduction in the MPW and CPW is the crystallization inhibition which is discussed in the next section in detail. Due to a much lower crystalline partition of the blends, it takes much less time to melt the crystalline part and to recrystallize, which also results in a smaller peak width. In which way the polymer crystallizes out is unfortunately difficult to estimate in this multicomponent system, although the formation of spherulites is a very likely approach [77].

**Figure 13:**
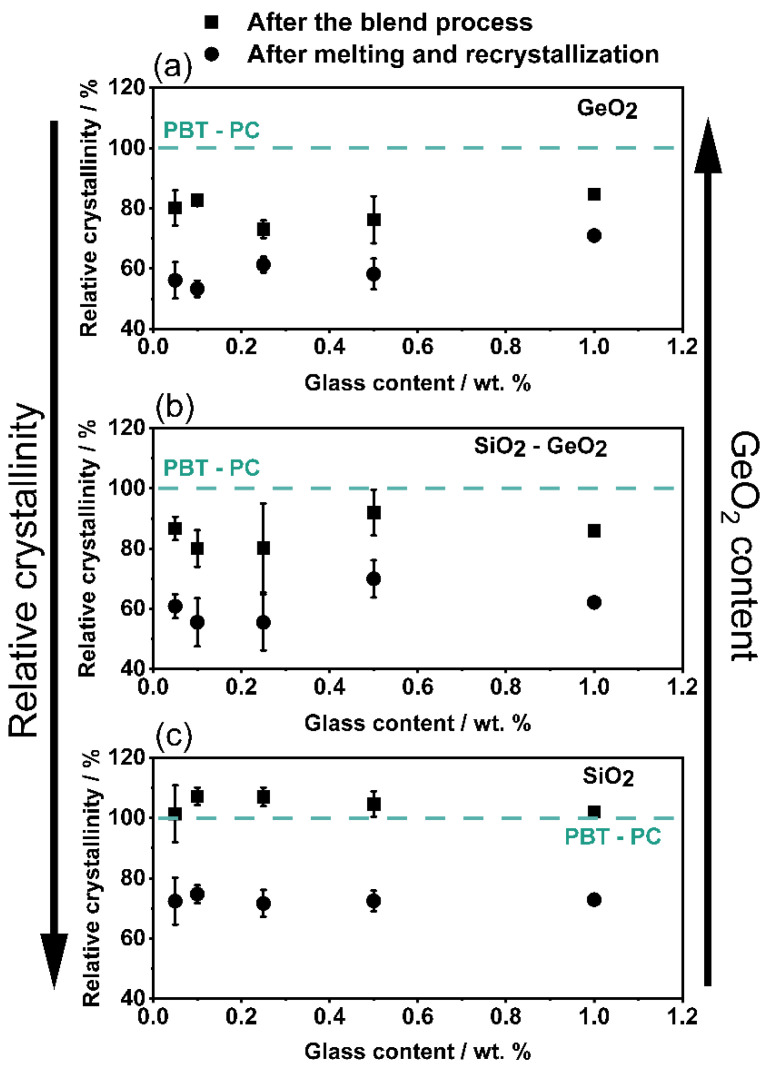
Influence of the glass content on the relative crystallinity of the PBT-PC composite. (**a**) GeO_2_; (**b**) SiO_2_-GeO_2_; (**c**) SiO_2._

The influence of the glass flakes on the crystallinity and therefore the proof of the glass flakes acting as crystallization inhibitor is of particular interest, as the crystallinity significantly determines the properties of the component after melting (whether in additive manufacturing or injection molding) as well as the miscibility of the two polymers. The relative crystallinities of the different formulations are shown in Figure 13. A crystallinity of 100% corresponds to the PBT-PC composite without glass filler. Looking at the results of the PBT-PC-GeO_2_ blends (Figure 13a), different results are obtained, where already after comminution a significantly lower crystallinity is observed compared to the original composite. Despite slight fluctuations, the relative crystallinity is around 80%, which means the samples containing GeO_2_ either lose their crystallinity during the co-comminution, which can happen through amorphization [53,78,79] which is eventually catalyzed by the presence of GeO_2_ as it is not occurring with pure SiO_2_, but also with the SiO_2_-GeO_2_ mixture. After melting and recrystallizing the PBT-PC-GeO_2_ samples, the crystallinity decreases further leaving a residual crystallinity of around 55–65% and therefore the lowest level of crystallinities in this study. Therefore the GeO_2_ inhibits the recrystallization of the material. In comparison with the pure GeO_2_ and the SiO_2_-GeO2 mixture, the crystallinity at the first melting (directly after comminution) of the SiO_2_-filled samples (Figure 13c) is unchanged compared to the pure PBT-PC after the blending process, which is a behavior known from functionalization with nano silica [80]. Only after melting and recrystallization is a reduction in crystallinity observed here, as also occurred in the samples measured here. The reason is, that particles, whether nano or anisotropic flakes, act as crystallization nuclei, this as well as a faster crystallization due to higher thermal energy transport significantly reduces the time it takes for a solid crystal structure to form. 

Both, the relative crystallinity after melting and crystallization shows clearly that the amorphous part of the blends are increased (when the crystallinity decreases the amorphous part increases) with the addition of GeO_2_., which means, the higher the GeO_2_ proportion in the additive, the more the crystallization in inhibited. Interestingly, the glass content itself has very less influence on the crystallinity which has to be investigated in future work. The measured variations are due to the always slightly different compositions of PBT-PC and in the samples. However, the SiO_2_ flakes prevent the PBT portion in the composite from crystallizing to some extent, so that (irrespective of the SiO_2_ amount) a crystallinity of about 70–75% of the initial value remains. This also fits with the results from the literature [80], where SiO_2_ nanoparticles as a flow aid can impair crystallization. The SiO_2_-GeO_2_ mix (50:50 wt./wt.), Figure 13b, shows behavior in between of the formulations with the pure glass flakes, which was to be expected. The experiments with the glass mix confirm that GeO_2_ has a significant effect on the crystallinity and crystallization of the composite, which has to be further investigated in future work.

## 4. Conclusions

It was proven in this study that GeO_2_ drastically influences the crystallinity and crystallization behavior of the mixtures in terms of reducing the relative crystallinity of the PBT-PC blend. As the relative crystallinity is reduced, the acceleration of crystallization is also reduced, which results in a more homogeneous amorphous solid dispersion. The reduction of crystallinity obtained during comminution and blending of the PBT-PC-glass composite is already prevented from recrystallizing due to the additivation and fluctuates between 75–80% for pure GeO_2_ as additive and 80–85% for SiO_2_-GeO_2_, whereas SiO_2_ additivated PBT-PC maintains its 100% crystallinity. After melting and recrystallizing, the crystallinity of the product decreases significantly in all experiments, leading to the conclusion, that form anisotropic particles themself are already reducing the crystallinity and GeO_2_ contents further intensifies this effect. This work further demonstrated that the production of PBT-PC-glass composites is possible within agitator bead mills, which opens up the possibility for the continuous production of such advanced materials in the future

## Figures and Tables

**Table 1:** Properties of PBT and PC raw material particles (self measured).

Property	PBT	PC
Particle size x_10,3_/µm	70	77
Particle size x_50,3_/µm	213	230
Particle size x_90,3_/µm	465	495
Density ρ_bulk_/g cm^−3^	1.33	1.22

**Table 2:** Characteristics of the glasses used for comminution experiments.

Material	Particle Size µm			Specific Surface Area m^2^ g^−1^	Density g cm^−3^
	x_10,3_	x_50,3_	x_90,3_		
SiO_2_	8	47	129	0.51	2.2717 +/− 0.0009
SiO_2_-GeO_2_	8	57	143	0.68	2.7771 +/− 0.0002
GeO_2_	6	36	99	0.49	3.7935 +/− 0.0003

**Table 3:** Overview of the set of parameters defining the standard experimental conditions for the glass flake production.

Parameter	Experimental Condition
Rotor revolution/min^−1^	2000
Rotational speed/m s^−1^	6.50
Grinding bead media	Yttria stabilized zirconia (5 wt. % Y_2_O_3_)
Grinding bead density/g cm^−3^	6.5
Grinding bead diameter d_GM_/mm	1.0
Solids concentration cw/wt. %	3.0
Grinding time/h	5
Temperature grinding chamber/°C	20 °C

**Table 4:** Overview of the set of parameters defining the standard experimental conditions for the composite production.

Parameter	Experimental Condition
Rotor revolution/min^−1^	2000
Rotational speed/m s^−1^	6.50
Grinding bead media	Yttria stabilized zirconia (5 wt. % Y_2_O_3_)
Grinding bead density/g cm^−3^	6.5
Grinding bead diameter d_GM_/mm	2.0
Solids concentration cw/wt. %	20.0
Grinding time/h	7
Temperature grinding chamber/°C	15 °C

**Table 5:** Plan of PBT-PC-Glass blends.

No	PBT-PC	SiO_2_	SiO_2_-GeO_2_	GeO_2_
		wt. %	wt. %	wt. %
1	15 g PBT 5 g PC	0.05		
2		0.1		
3		0.25		
4		0.5		
5		1.0		
6	15 g PBT 5 g PC		0.05	
7			0.1	
8			0.25	
9			0.5	
10			1.0	
11	15 g PBT 5 g PC			0.05
12				0.1
13				0.25
14				0.5
15				1.0

**Table 6:** Main IR absorption bands for SiO_2_- and GeO_2_-based glasses.

Bond	Type	Wavenumber/cm^−1^	Literature
Ge-O-Ge	Bending vibration	300–470	[59]
Si-O-Si	Asymmetric vibration	450	[59]
Ge-O-Ge	Symmetric vibration	515–565	[56,58]
Si-O-Ge	Stretching vibration	660	[57,59]
Si-O-Si	Symmetric vibration	800	[65]
Ge-O-Ge	Asymmetric vibration	820–890	[58,66]
Si-O-Ge	Stretching vibration	990	[57,59]
Si-O-Si	Stretching vibration	1050–1200	[59,67]

## Data Availability

The data that support the findings of this study are available from the corresponding author, upon reasonable request.

## Supplementary Materials

The following supporting information can be downloaded at: https://www.mdpi.com/article/10.3390/polym14214555/s1, Figure S1: Surface area (a) and powder density (b) change of the utilized glasses with increasing comminution time; Figure S2: Particle size distributions (a) sum distribution Q3 and (b) density distribution q3; Figure S3: Multi-point BET measurement curves; (A) GeO2 glass, (B) SiO2 glass and (C) the 50:50 wt/wt mixture; Figure S4: Example DSC curves of PBT-PC-GeO2 (a) 0.05 wt.%; (b) 0.1 wt.%; (c) 0.25 wt.%; (d) 0. 5 wt.%; (e) 1.0 wt.%; Figure S5: Example DSC curves of PBT-PC-SiO2-GeO2 (a) 0.05 wt.%; (b) 0.1 wt.%; (c) 0.25 wt.%; (d) 0. 5 wt.%; (e) 1.0 wt.%; Figure S6: Example DSC curves of PBT-PC-SiO2 (a) 0.05 wt.%; (b) 0.1 wt.%; (c) 0.25 wt.%; (d) 0. 5 wt.%; (e) 1.0 wt.%.
