# Supplementary material for: Control of Crystallization of PBT-PC Blends by Anisotropic SiO2 and GeO2 Glass Flakes
Source: Polymers (Basel). 2022 Oct 27;14(21):4555. doi: 10.3390/polym14214555 (PMC9658842; doi:10.3390/polym14214555)
Supplement: Supplementary file 1 [file polymers-14-04555-s001.zip › polymers-1993038-supplementary.pdf]

Supporting Information

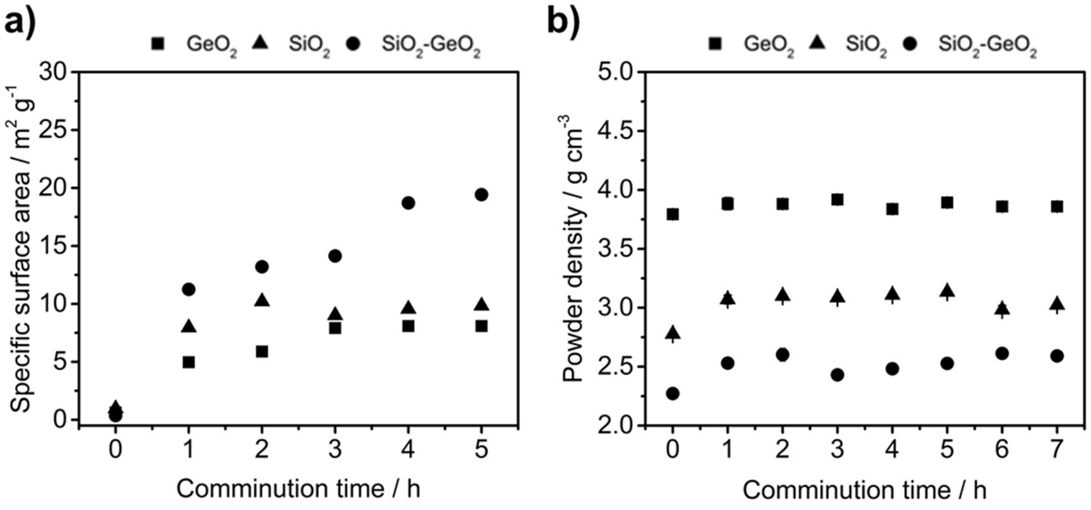

Figure S1. Surface area (a) and powder density (b) change of the utilized glasses with increasing comminution time.

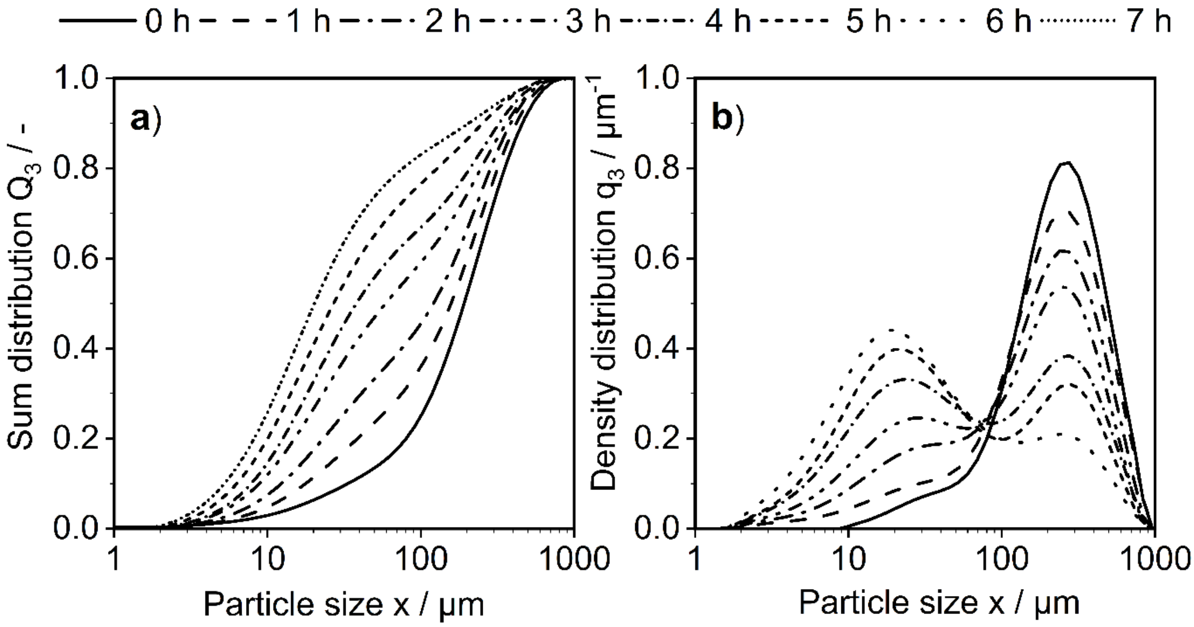

Figure S2. Particle size distributions (a) sum distribution Q_3 and (b) density distribution q_3

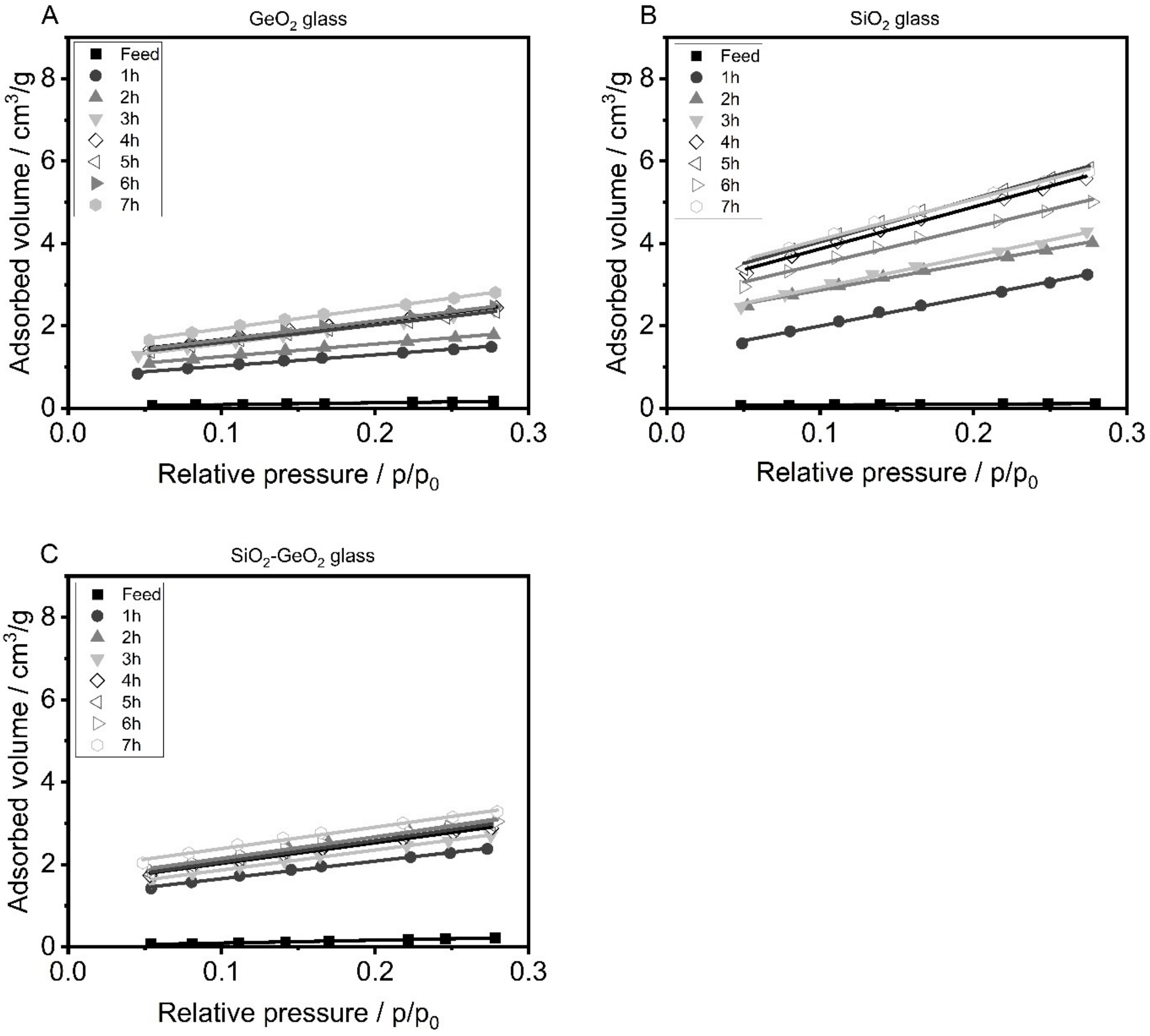

Figure S3. Multi-point BET measurement curves; (A) GeO_2 glass, (B) SiO_2 glass and (C) the 50:50 wt/wt mixture

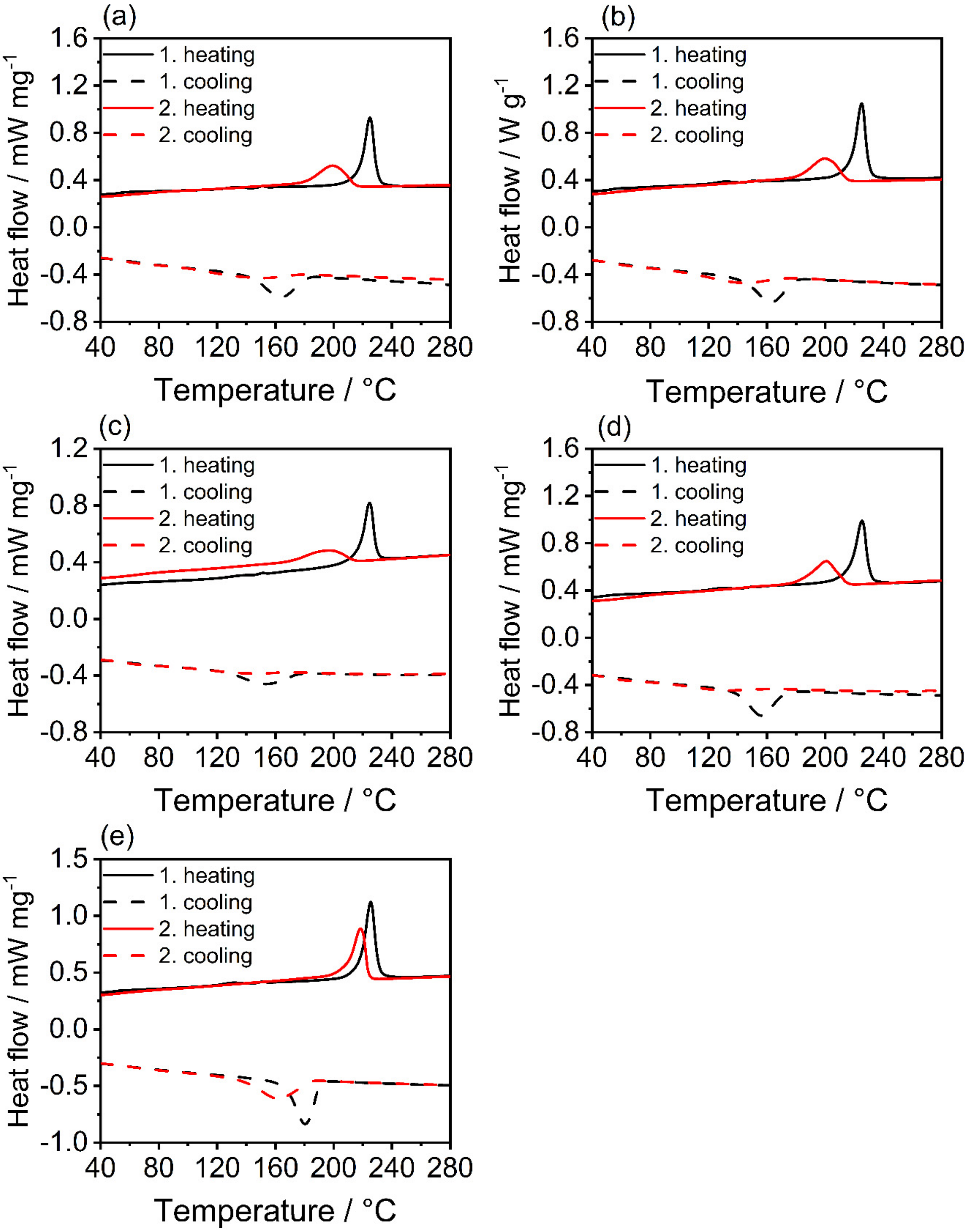

Figure S4. Example DSC curves of PBT-PC-GeO_2 (a) 0.05 wt.%; (b) 0.1 wt.%; (c) 0.25 wt.%; (d) 0. 5 wt.%; (e) 1.0 wt.%;

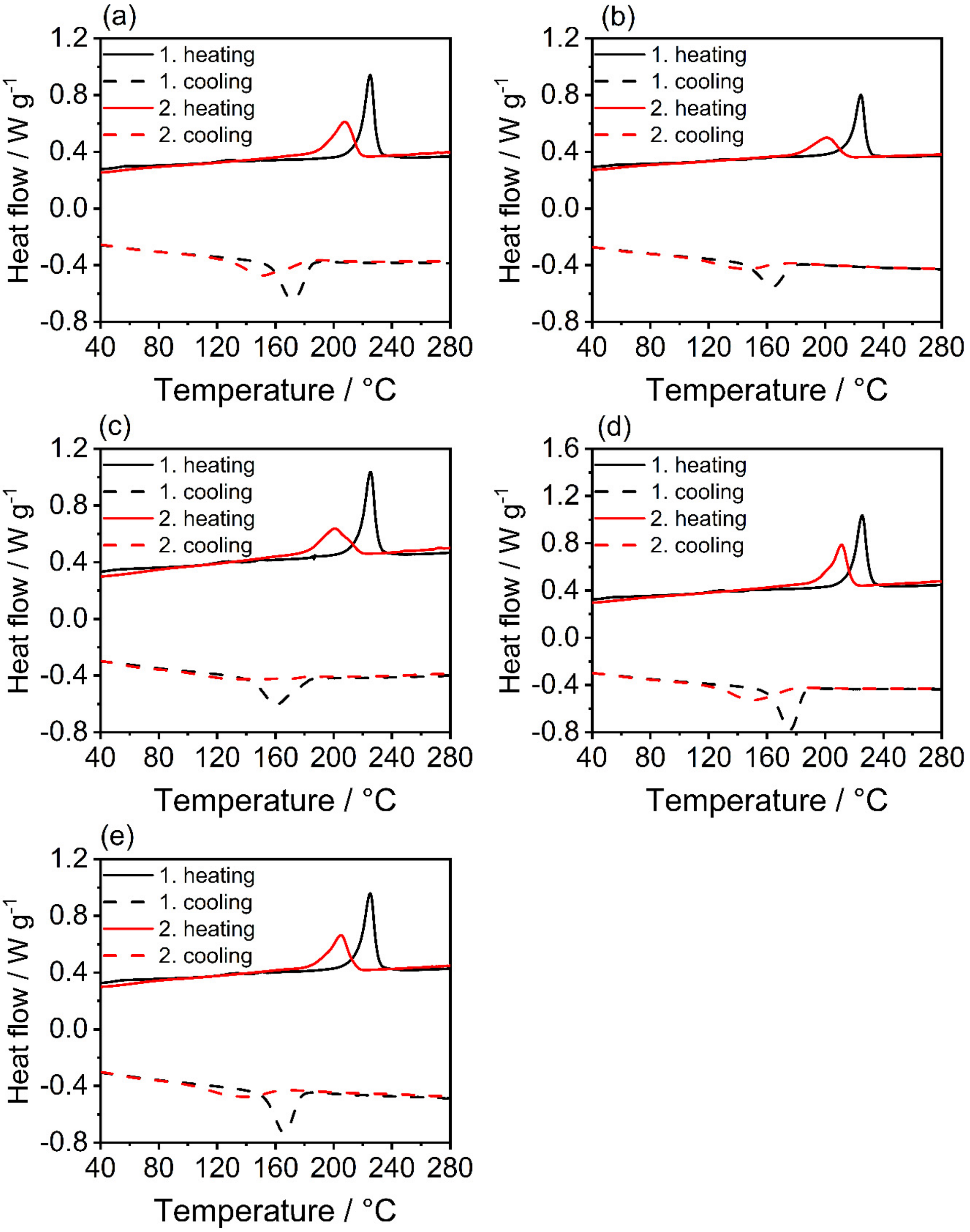

Figure S5. Example DSC curves of PBT-PC-SiO_2-GeO_2 (a) 0.05 wt.%; (b) 0.1 wt.%; (c) 0.25 wt.%; (d) 0. 5 wt.%; (e) 1.0 wt.%;

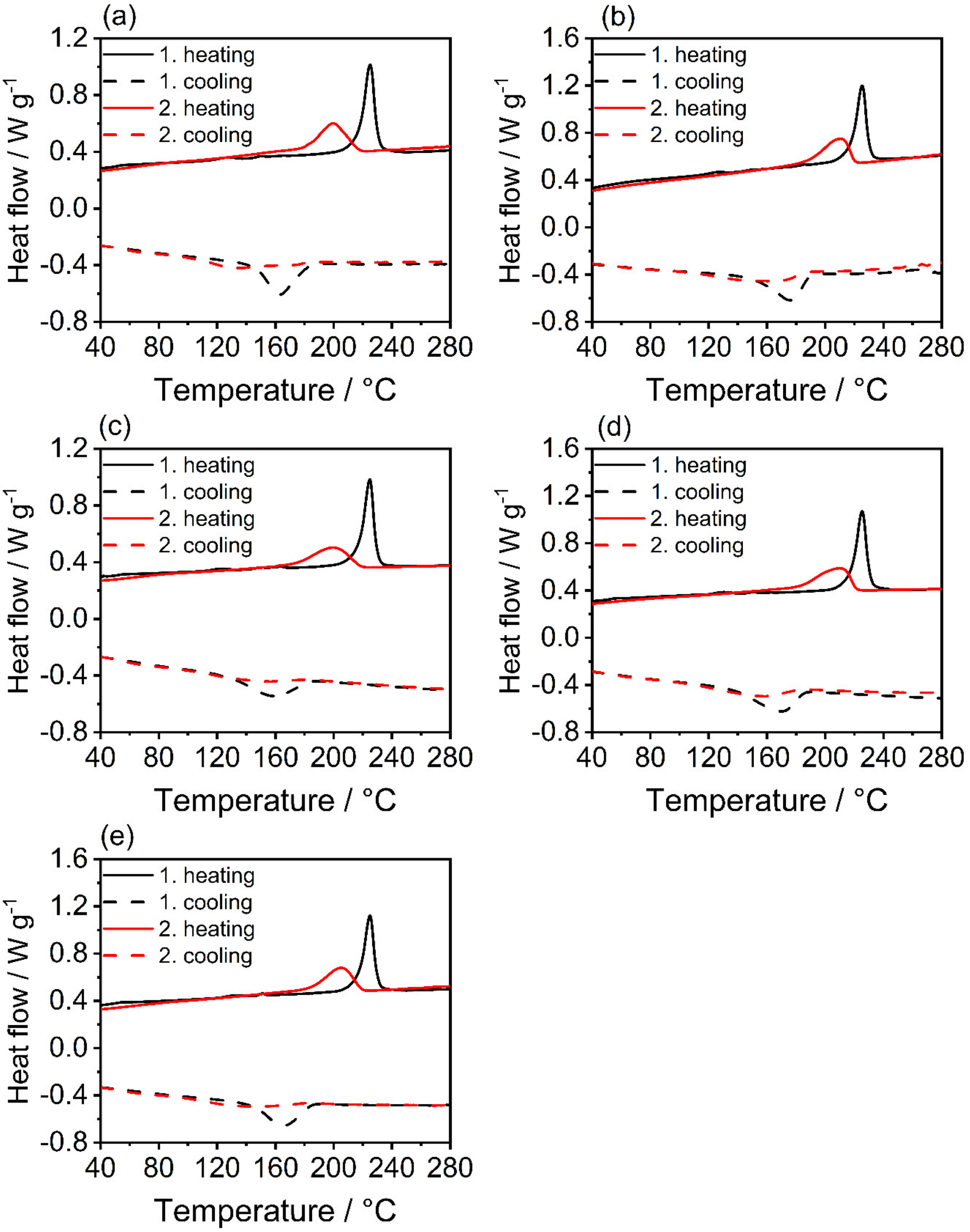

Figure S6. Example DSC curves of PBT-PC-SiO_2 (a) 0.05 wt.%; (b) 0.1 wt.%; (c) 0.25 wt.%; (d) 0. 5 wt.%; (e) 1.0 wt.%;
